# Income Inequality and Happiness: An Inverted U-Shaped Curve

**DOI:** 10.3389/fpsyg.2017.02052

**Published:** 2017-11-24

**Authors:** Zonghuo Yu, Fei Wang

**Affiliations:** ^1^School of Psychology, Jiangxi Normal University, Nanchang, China; ^2^Department of Psychology, School of Social Sciences, Tsinghua University, Beijing, China

**Keywords:** income inequality, Gini coefficient, happiness, curvilinear model, inflection point

## Abstract

Numerous studies agree that income inequality, rather than absolute income, is an important predictor of happiness. However, its specific role has been controversial. We argue that income inequality and happiness should exhibit an inverted U-shaped relationship due to the dynamic competing process between two effects: when income inequality is relatively low, the *signal effect* will be the dominating factor, in which individuals feel happy because they consider income inequality as a signal of social mobility and expect upward mobility; however, if income inequality level increases beyond a critical point, the *jealousy effect* will become the dominating factor, in which individuals tend to be unhappy because they are disillusioned about the prospect of upward mobility and jealous of their wealthier peers. This hypothesis is tested in a longitudinal dataset on the United States and a cross-national dataset on several European countries. In both datasets, the Gini coefficient (a common index of a society’s income inequality) and its quadratic term were significant predictors of personal happiness. Further examinations of the quadratic relationships showed that the signal effect was only presented in the European data, while the jealousy effect was presented in both datasets. These findings shed new light on our understanding of the relationship between income inequality and personal happiness.

## Introduction

“The lord of a state or a family, concerns himself not with scarcity but rather with uneven distribution… For where there is even distribution, there is no poverty.”

– Confucius

The relationship between wealth and subjective well-being is a major issue in social science research. Extant studies have found complex relationships between income and happiness. For example, there is evidence that money does not always buy happiness. After material wealth reaches a certain level, its further increase no longer promotes happiness (Easterlin, 1973, 1974, 1995). This conclusion, also called the “Easterlin paradox” (i.e., more wealth does not lead to more happiness), may originate from individuals’ satisfaction with their lives being rather affected by their spontaneous comparison between themselves and others. According to the social comparison theory (Festinger, 1954), there are two types of social comparisons: upward comparison, which involves comparing oneself with those doing better, and downward comparison, which involves comparing oneself with those doing worse. Critically, the proclivity of upward comparison is significantly stronger than for the downward comparison (Ferrer-i-Carbonell, 2005; Boyce et al., 2010). As a result, even if their absolute income increases, individuals would be still more likely to compare themselves to those richer, which may deteriorate their subjective well-being.

Consequently, we can expect the income gap between the rich and poor to be a better predictor of happiness. Indeed, empirical studies have shown a significant association between the income gap (indexed by the Gini coefficient) and happiness (Brockmann et al., 2009; Oishi et al., 2011; Asadullah and Chaudhury, 2012). However, the shape of the income inequality-happiness function is still controversial, as the empirical results have been mixed. While some studies show income inequality and happiness to have a negative relationship (Alesina et al., 2004; Oishi et al., 2011; Verme, 2011), others suggest a positive association (Clark, 2006; Caporale et al., 2009), and some find them unrelated (Helliwell, 2003).

In the current study, we aimed to resolve this inconsistency by exploring a new possibility: the relationship between income inequality and happiness is not linear, but curvilinear. Our hypothesis is based on previous theories that suggest the income inequality-happiness relationship is mainly affected by two competing psychological processes: the jealousy and signal effects (Senik, 2004, 2008). The jealousy effect suggests that, when income inequality is high, individuals tend to be unhappy because they are jealous of their wealthier peers (Senik, 2008). By contrast, the signal effect posits that individuals might consider income inequality a signal of social mobility and expect upward mobility (Senik, 2004, 2008). Critically, we propose that both effects manifest in the presence of an income gap, and push the income inequality-happiness relationship into opposite directions. However, their relative strength depends on how large the income inequality actually is. When it is in the low range, it is easier for individuals to climb the social ladder, and the signal effect will be the main determinant in encouraging individuals to be more hopeful about their future. At this stage, a positive relationship is expected between income inequality and happiness, because a higher income gap means higher possible status. However, as the income inequality level increases beyond a critical point, the top rungs of the social ladder become almost unreachable for most members of the society. As a result, individuals become less hopeful regarding upward mobility and, as the jealousy effect becomes the dominating factor, happiness decreases, while the income gap increases.

In summary, the dynamic competition between the jealousy and signal effects would convert the income inequality-happiness relationship into an inverted U-shaped curve. Previous studies have failed to identify this curvilinear relationship, because they mostly evaluated the fit of linear models. In the current research, we evaluate the fit of curvilinear models for the income inequality-happiness relationship based on two large datasets.

## Study 1: Longitudinal Data on the United States

In Study 1, we tracked how personal happiness changes as a function of a country’s income inequality in a longitudinal dataset collected in the United States during 1972–2010.

### Methods

#### Sample

Data on personal income and happiness were obtained from the General Social Survey (GSS), a large-scale sociological survey on the attitudes, behaviors, and attributes of the contemporary Americans conducted by the research institute National Opinion Research Center (NORC) at the University of Chicago. It was launched in 1972 and was conducted almost annually until 1994. Since 1994, it has been conducted in even years only. The GSS contains a wide range of measures, covering topics such political attitudes and psychological well-being (for more details on the GSS, see: http://www.gss.norc.org). Here, we used GSS data on the household income and happiness for 50,357 United States residents during 1972–2010. During this period, 28 surveys were administered, but the data for 1978, 1983, and 1986 do not contain health information, which is an important covariate to predicting happiness. Therefore, we used the data from the remaining 25 years. Students were excluded from the sample, as they typically do not have an independent income. Respondents who identified themselves as other than white or black were also excluded, since previous research has shown that race may affect happiness, and the size of these other racial samples were too small to make any comparison meaningful. However, the main results remained consistent even when respondents of all races were included in the analysis (see Supplementary Tables S16–S18). Missing values were dealt with using a list-wise deletion approach. The final sample size was 31,271. The yearly sample size varied from 690 in 2004 to 2,207 in 1998. More details on the descriptive statistics of all pertinent measured variables are shown in the Supplementary Materials.

In the GSS dataset, from 1972 to 2010, the annual income of United States households increased from USD 28,531.3391 to USD 32,882.1141. Although there were occasional falls, an overall rising trend was registered. Moreover, during the same period, the Gini coefficient also rose from 0.36 in 1972 to 0.46 in 2010 (for more details, see Supplementary Tables S1, S2).

#### Measures

##### Income inequality

Information on income distribution (Gini coefficient) was obtained from the United States Census Bureau. The Gini coefficient (sometimes expressed as the Gini ratio or normalized Gini index) is a measure of statistical dispersion representing the income or wealth distribution of a nation’s residents. It is a widely used measure of income inequality in a country, with scores ranging from 0 to 1.

##### Income

The income level was measured in terms of household income per capita.

##### Happiness

A three-point scale (3 = *very happy*, 2 = *pretty happy*, 1 = *not too happy*) was used to measure participants’ level of happiness.

##### Control variables

Individual-level control variables included age, education, gender, living with children or not, ethnicity, employment status, marital status, health status, and household size. Country-level control variables included year of data collection and per capita gross domestic product (GDP) growth rate. The results were consistent, even when several covariates were excluded from the model (see Supplementary Table S18).

#### Model Specifications

Mixed-effects modeling (Raudenbush and Bryk, 2002) was performed on the above described United States data. The unit of analysis at level 1 was the individual respondent, level 1 measures including demographic variables such as gender, age, health status, education level, household size, whether the respondent had children or not, marriage status, income, and occupation. Several previous studies have found an inverted U-shaped relationship between age and happiness. However, since we excluded students from the analysis, we did not include the quadratic term of age in the model. Moreover, household annual income was measured as a continuous variable in the GSS. Our results remained constant when we treated income in the United States data as a continuous variable or recoded income into five ordinal categories. The demographic variables were included in the model as control measures.

The units of analysis at level 2 were the survey years in the GSS. The GSS is a pooled cross-sectional dataset, with a different cross-sectional sample for each annual survey. The Gini coefficient, a level 2 measure, was the focal predictor of the model. We also included the GDP growth rate as a level 2 control variable. In the analysis, the year of the survey was also included as a control variable.

Demographic and income variables were controlled in the first-level analysis and GDP growth rates were controlled in the second level. The GSS model further controlled surveyed time. Critically, both the Gini coefficient and its quadratic terms were entered as predictors in the second level as to model the curvilinear relationship between the Gini and happiness (for more details about the model specifications, see the Supplementary Materials).

### Results

As previously mentioned, during 1972–2010, the surveyed annual income of United States households increased from USD 28,531.3391 to USD 32,882.1141. According to psychophysical theories, happiness can be better predicted by the logarithm of income (Kahneman and Deaton, 2010). Therefore, we performed a logarithmic transformation of household income, and the transformed income was significantly correlated with happiness (*r* = -0.403, *p* = 0.046). The correlation between the Gini coefficient and happiness was also significant (*r* = 0.445, *p* = 0.022).

**FIGURE 1:**
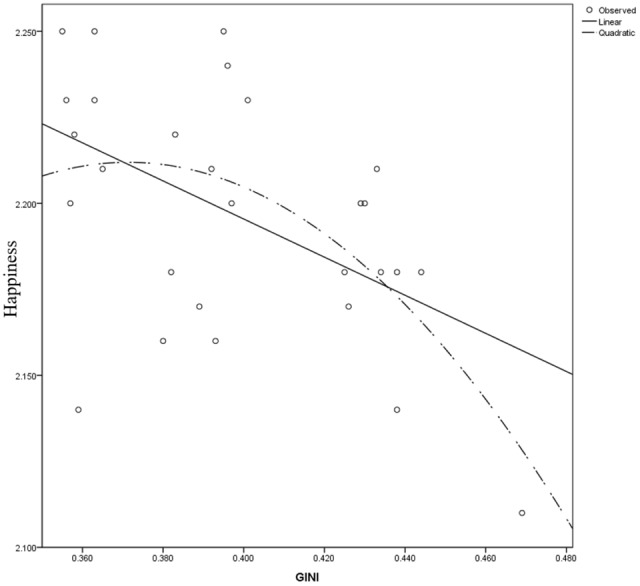
Gini-happiness curve for the United States.

In the mean happiness model based on the GSS dataset, after controlling for demographics, the surveyed year was not a significant predictor of happiness (regression coefficient = -12.571, *t*_(20)_ = -1.045). Gini coefficient’s regression coefficient was 26.023, *t*_(20)_ = 2.557, *p* = 0.019, and its quadratic term’s regression coefficient -36.527, *t*_(20)_ = -2.872, *p* = 0.010. Combining these two effects revealed an inverted U-shaped relationship between the Gini coefficient and happiness (**Figure 1**). For an individual with average income, the inflection point of the Gini-happiness relationship was 0.356^1^. GDP growth rate’s regression coefficient was 0.015, *t*_(20)_ = 2.461, *p* = 0.023. We further tested whether the relationship between the Gini coefficient and happiness was significantly positive before the inflection point, and significantly negative after the inflection point (Simonsohn, 2017). Because the sample size at level 2 was too small to be splitted into two parts, here we used single-level models. Before the inflection point, Gini coefficient’s regression coefficient was -4.827, *t*_(2583)_ = -0.195, *p* = 0.845, and after the inflection point, -0.749, *t*_(22677)_ = -6.411, *p* < 0.001. Therefore, only the second part of our hypothesis (the jealousy effect) was significant. However, there were only 2 years’ data (1973 and 1974) before the inflection point, which might hinder the detection of the signal effect.

In the model of slopes of household income and happiness, the intercept was -0.284, *t*_(23)_ = -1.096, *p* = 0.285. Gini coefficient’s regression coefficient was 1.890, *t*_(23)_ = 2.991, *p* = 0.007. Therefore, although an individual’s absolute income level effect on happiness is not significant, the relationship becomes stronger as the Gini coefficient increases.

## Study 2: Cross-National Data On European Countries

In Study 2, we further tested the curvature relationship between the Gini coefficient and happiness in a large dataset from a cross-national survey on European countries.

### Methods

#### Sample

Data on personal happiness from European countries were obtained from the European Social Survey (ESS), a large-scale sociological survey similar to the GSS, initiated by the European Science Foundation, which has been conducted biannually since 2002 in several European countries. We included data from generalized Western European countries, namely Austria, Belgium, Germany, Greece, Ireland, Italy, Luxembourg, Netherlands, Portugal, Slovenia, Spain, and the United Kingdom. Scandinavian countries (Denmark, Finland, Norway, Iceland, and Sweden) were excluded from the analysis because their welfare systems are significantly different from those of Western Europe (Esping-Andersen, 1990, 1996). In the United States and Western European countries, welfare is closely related to a citizen’s personal income, and the market plays an important role in it. By contrast, in Scandinavian countries, most citizens are included in a state welfare system, and the roles of individuals and the market are much more limited. Therefore, including these countries may have distorted the relationship between income inequality and happiness (results including these countries are presented in Supplementary Tables S22–S24). Analysis was restricted to the ESS data collected in 2002, 2004, and 2006, because accurate data on income distribution for the analyzed European countries were difficult to obtain from 2008 onward. The effective sample sizes for the three considered surveys were 12,297 in 2002, 11,480 in 2004, and 11,575 in 2006. More details about the summary statistics for the pertinent measured variables are shown in the Supplementary Materials section.

#### Measures

##### Income inequality

We obtained data on European countries’ Gini coefficients from the World Income Inequality Database.

##### Income

The income level was measured as household total net income.

##### Happiness

In ESS, respondents used an 11-point scale (0 = extremely unhappy to 10 = extremely happy) to rate their levels of happiness.

##### Control variables

Individual-level control variables included age, education, gender, living with children or not, employment status, marital status, and self-rated health. The country-level control variable was the GDP growth rate.

#### Model Specifications

In the ESS data, happiness (dependent variable) is a continuous variable and income level (independent variable) an ordinal variable with 12 categories. To avoid creating too many dummy variables and also facilitate comparison with the United States results, we divided the sample into five income categories and created a set of dummy variables with the middle category as reference. In these models, the first level of analysis was the individual. The unit of the second level of analysis in ESS was the country. Demographic and income variables were controlled in the first level and GDP growth rates in the second level. The Gini coefficient and its quadratic term were entered as predictors in the second level to model the curvilinear relationship between the Gini coefficient and happiness. For more details on the model specifications see the Supplementary Materials section.

### Results

**FIGURE 2:**
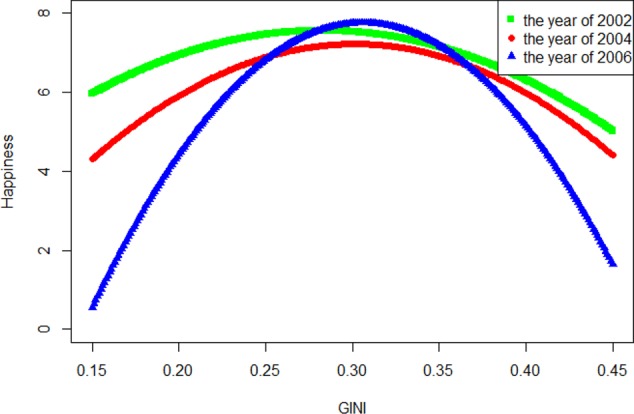
Gini-happiness curve in Western European countries (2002–2006).

**FIGURE 3:**
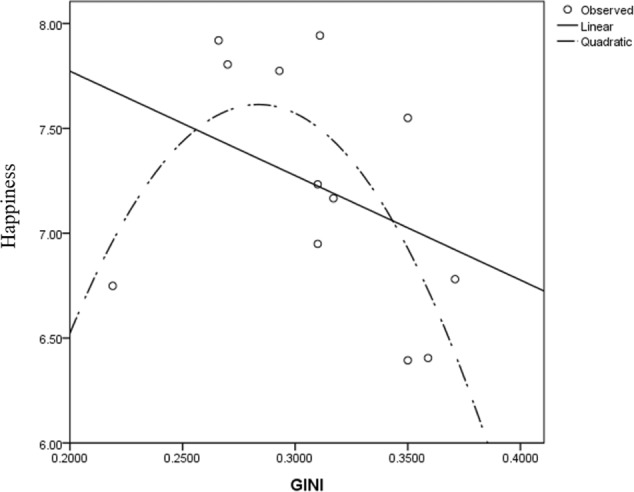
Scatter plot of GINI-happiness for ESS, 2002.

**FIGURE 4:**
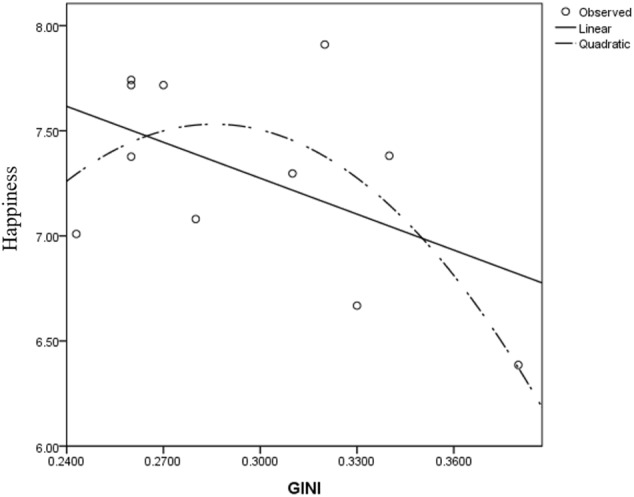
Scatter plot of GINI-happiness for ESS, 2004.

**FIGURE 5:**
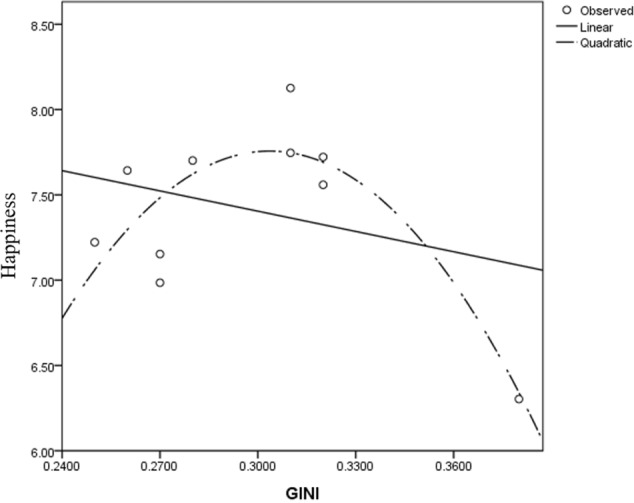
Scatter plot of GINI-happiness for ESS, 2006.

In the ESS dataset, the models were estimated separately for the three waves (2002, 2004, and 2006; **Figures 3**–**5**). Taking the 2006 model as an example, after controlling for demographics and GDP growth rates, in the average happiness model, the Gini coefficient’s regression coefficient was 180.797, *t*_(6)_ = 8.383, *p* < 0.001. Its quadratic term regression coefficient was -295.310, *t*_(6)_ = -8.450, *p* < 0.001. Combining these two effects revealed an inverted U-shaped Gini-happiness relationship (**Figure 2**). The Gini coefficient at the inflection point of this curve was 0.286. Because the sample size at level 2 was too small to be splitted into two parts, here we used single-level models to further test the relationship between the Gini coefficient and happiness. Before the inflection point, the Gini regression coefficient was 6.279, *t*_(6142)_ = 2.777, *p* = 0.006 and -42.546, *t*_(4391)_ = -8.591, *p* < 0.001 after the inflection point.

The 2002 and 2004 models yielded similar results. Both the Gini and its quadratic term’s regression coefficients were significant, resulting in an inverted U-shaped Gini-happiness curve. The inflection points for these 2 years were 0.301 and 0.306, respectively. For the 2002 model, before the inflection point, the Gini regression coefficient was 10.541, *t*_(3513)_ = 9.411, *p* < 0.001 and -11.513, *t*_(7650)_ = -10.615, *p* < 0.001 after the inflection point. For the 2004 model, before the inflection point, the Gini regression coefficient was 15.819, *t*_(3476)_ = 2.621, *p* = 0.009 and -4.711, *t*_(7342)_ = -6.618, *p* < 0.001 after the inflection point. Considering that the different waves of ESS data differed only slightly for the countries with complete data, these patterns are quite robust. The results were consistent, even when several covariates were excluded from the model (see Supplementary Tables S19–S21).

## General Discussion

A quadratic relationship between income inequality and happiness was found for yearly variations of happiness within the same country (the United States) and across European countries for the same year. In the United States data, the quadratic relationship was primarily due to the predominance of the jealousy effect for the comparatively high range of income inequality, while in the European data, both the signal and jealousy effect were presented, resulting in an overall inverted U-shaped relationship. Income redistribution can be viewed as a way to optimize efficiency and fairness toward economic growth. As excessive income inequality may impair fairness, excessive economic egalitarianism may reduce efficiency. Therefore, the results of this study suggest that, before a critical level of income inequality is reached, rising income inequality may be accompanied by a higher level of happiness, probably because the social comparison of aspiring individuals with their richer co-nationals promotes expectations that the income gap can be closed and offers a sense of financial optimism. However, once the income gap seems too wide to cross due to rising income inequality, more aspiring individuals may replace their upward mobility dream with despair and feel jealous of the rich.

The values of the inflection points vary across countries, with higher values in the United States than Western Europe. Compared to Western Europeans, Americans seem to maintain their aspirations for upward mobility and do not show the jealousy effect until reaching a high level of income inequality. This high inflection point in the United States may partly result from the popular belief in the “American dream.” Relative to Europe, the United States has a higher level of income inequality and a lower level of intergenerational mobility (Organisation for Economic Co-operation and Development, 2010). However, also compared to Europeans, the Americans believe more strongly they live in a high mobility society and disagree more strongly that poverty is stationary (Alesina et al., 2001, 2004). Furthermore, Americans may be more willing to accept challenges and have greater ambition (Alesina et al., 2004). The finding that the inflection point is lower in Europe can also explain the stronger popular support for income redistribution policies compared to the United States, despite the lower level of income equality in Europe (Alesina et al., 2004).

However, it should be noted that the inverted U-shaped curve was not valid after including the Scandinavian countries (Supplementary Tables S22–S24), suggesting that social institutions may play an important role in the relationship between the Gini coefficient and happiness. Therefore, more research is needed to test the generalizability of the current results outside the United States and Western European countries. Another limitation of the current research is that it does not directly test the proposed psychological mechanisms underlying the inverted U shape. Future studies thus can manipulate these mechanisms in experiments or test them using a simulation approach. Nonetheless, our results indicate that the relationship between income inequality and happiness is more complex than previous studies have assumed.

## Author Contributions

ZY: developed and validated the theoretical model, data preparation and analysis, manuscript writing and revision. FW: expanded the theoretical model, manuscript writing and revision. ZY designed the research and analyzed the data with the assistance of FW. Both authors wrote the manuscript and approved the final version of the manuscript for submission.

## Conflict of Interest Statement

The authors declare that the research was conducted in the absence of any commercial or financial relationships that could be construed as a potential conflict of interest.

## Footnotes

1 The number seems a little lower than the inflection point of **Figure 1**, which was drawn without controlling any variable.
