# Supplementary material for: Income Inequality and Happiness: An Inverted U-Shaped Curve
Source: Front Psychol. 2017 Nov 24;8:2052. doi: 10.3389/fpsyg.2017.02052 (PMC5705943; doi:10.3389/fpsyg.2017.02052)
Supplement: Supplementary file 1 [file Table_1.DOCX]

# Supplemental material

Table S1. Summary Statistics for All Variables, GSS 1972-2010

| Level-1: individual (*N*=31271) | | % or Mean |
| --- | --- | --- |
| Happiness | |  |
| very happy | | 31.98 |
| pretty happy | | 55.74 |
| not too happy | | 12.28 |
| Gender | |  |
| female | | 54.96 |
| male | | 45.04 |
| Employment status | |  |
| Full time | | 53.24 |
| Part-time | | 10.51 |
| temporarily unemployed | | 2.28 |
| Unemployed | | 3.21 |
| Retired | | 12.99 |
| keeping house | | 17.77 |
| Marital status | |  |
| single | | 17.61 |
| married | | 57.39 |
| widowed | | 9.47 |
| divorced | | 12.06 |
| separated | | 3.47 |
| Race/Ethnicity | |  |
| black | | 14.06 |
| white | | 85.94 |
| Living with child(ren) | |  |
| no | | 25.77 |
| yes | | 74.23 |
| Self-rated health | |  |
| unhealthy | | 4.90 |
| healthy | | 95.10 |
| Age | | 45.64(17.01) |
| Years of schooling | | 12.67(3.17) |
| annual household per capita income | | 13877.05(13924.08) |
| Level 2: Survey Year (*N*=25) |  | |
| Gini | 0.40 (0.03) | |
| GDP per capita annual growth (%) | 2.87 (2.32) | |

Note: Percentages for categorical variables and means for continuous variables are reported, and numbers in parentheses are.

Table S2. Relationship of Happiness with Measured Variables, GSS 1972-2010, (%, *N*=31271)

|  | very happy | pretty happy | not too happy |
| --- | --- | --- | --- |
| Gender |  |  |  |
| female | 32.42 | 55.15 | 12.43 |
| male | 31.44 | 56.47 | 12.09 |
| Employment status |  |  |  |
| Full time | 31.74 | 58.06 | 10.20 |
| Part-time | 30.30 | 57.01 | 12.69 |
| temporarily unemployed | 29.45 | 54.84 | 15.71 |
| Unemployed | 15.85 | 53.84 | 30.31 |
| Retired | 35.43 | 51.44 | 13.12 |
| keeping house | 34.40 | 51.66 | 13.94 |
| Marital status |  |  |  |
| single | 21.33 | 63.07 | 15.60 |
| married | 40.28 | 51.98 | 7.73 |
| widowed | 23.84 | 56.45 | 19.72 |
| divorced | 19.20 | 62.33 | 18.46 |
| separated | 15.38 | 55.89 | 28.73 |
| Race |  |  |  |
| black | 21.91 | 56.30 | 21.79 |
| white | 33.63 | 55.65 | 10.72 |
| Living with child(ren) |  |  |  |
| no | 28.93 | 59.44 | 11.63 |
| yes | 33.04 | 54.46 | 12.50 |
| Self-rated health |  |  |  |
| unhealthy | 17.62 | 47.85 | 34.53 |
| healthy | 32.72 | 56.15 | 11.13 |
| Age group |  |  |  |
| 18-29 | 28.01 | 59.65 | 12.34 |
| 30-39 | 31.41 | 57.50 | 11.09 |
| 40-49 | 30.88 | 57.06 | 12.06 |
| 50-59 | 33.72 | 53.00 | 13.28 |
| 60-69 | 36.38 | 51.25 | 12.37 |
| 70 and above | 35.11 | 51.44 | 13.45 |
| Education |  |  |  |
| 0-11 years | 29.12 | 52.58 | 18.30 |
| 12 years | 30.95 | 56.99 | 12.06 |
| 13-16 years | 33.12 | 57.24 | 9.64 |
| 17-20 years | 38.57 | 54.26 | 7.17 |
| Income group |  |  |  |
| 1st quartile | 23.64 | 55.65 | 20.70 |
| 2nd quartile | 29.27 | 56.74 | 13.98 |
| 3rd quartile | 33.35 | 56.69 | 9.95 |
| 4th quartile | 34.34 | 56.76 | 8.90 |

| Table S3. Correlation Matrix for Yearly Averages of Happiness and Income, GSS 1972-2010 | | | | | | | | | | | |
| --- | --- | --- | --- | --- | --- | --- | --- | --- | --- | --- | --- |
|  | 1 | 2 | 3 | 4 | 5 | 6 | 7 | 8 | 9 | 10 | 11 |
| 1 |  |  |  |  |  |  |  |  |  |  |  |
| 2 | .864** |  |  |  |  |  |  |  |  |  |  |
| 3 | .820** | .974** |  |  |  |  |  |  |  |  |  |
| 4 | .572** | .661** | .614** |  |  |  |  |  |  |  |  |
| 5 | .724** | .801** | .791** | .916** |  |  |  |  |  |  |  |
| 6 | 0.102 | 0.290 | 0.223 | .820** | .554** |  |  |  |  |  |  |
| 7 | .864** | .674** | .682** | .501* | .688** | -0.050 |  |  |  |  |  |
| 8 | .791** | .866** | .810** | .844** | .884** | .553** | .588** |  |  |  |  |
| 9 | .870** | .712** | .723** | .463* | .659** | -0.081 | .972** | .580** |  |  |  |
| 10 | .455* | 0.150 | 0.163 | -0.053 | 0.108 | -.403* | .592** | 0.028 | .558** |  |  |
| 11 | -0.259 | -0.326 | -0.242 | 0.094 | -0.032 | 0.166 | 0.020 | -0.266 | 0.032 | 0.129 |  |
| Note:1=Gini coefficient,2=Mean of annual household per capita income,3=Standard deviation of annual household per capita income,4=Mean of annual household income,5=Standard deviation of annual household income,6=Mean of annual household income (Logarithm),7=Standard deviation of annual household income (Logarithm),8=Mean of annual household per capita income (Logarithm),9=Standard deviation of annual household per capita income (Logarithm),10=Mean of happiness,11=Standard deviation of happiness | | | | | | | | | | | |

Table S4. Summary Statistics for All Measured Variables, Western European Countries, ESS 2002

| Variables | % or Mean |  | Happiness | |
| --- | --- | --- | --- | --- |
|  |  |  | Mean | Std. Dev. |
| Level-1: individual (*N*=12297) |  |  |  |  |
| Gender |  |  |  |  |
| Female | 49.26 |  | 7.14 | 2.11 |
| Male | 50.74 |  | 7.28 | 1.88 |
| Employment status |  |  |  |  |
| Employed | 54.33 |  | 7.39 | 1.80 |
| Unemployed | 5.39 |  | 6.21 | 2.38 |
| Retired | 25.41 |  | 7.04 | 2.15 |
| Home Making | 14.87 |  | 7.22 | 2.11 |
| Marital status |  |  |  |  |
| Never married | 19.47 |  | 7.12 | 1.95 |
| Married | 61.89 |  | 7.42 | 1.88 |
| Widowed | 10.86 |  | 6.45 | 2.41 |
| Divorced | 6.20 |  | 6.90 | 2.04 |
| Separated | 1.59 |  | 6.33 | 2.07 |
| Living with child(ren) |  |  |  |  |
| No | 58.01 |  | 7.15 | 2.02 |
| Yes | 41.99 |  | 7.29 | 1.96 |
| Self-rated health |  |  |  |  |
| Unhealthy | 7.64 |  | 5.64 | 2.49 |
| Healthy | 92.36 |  | 7.34 | 1.89 |
| Education |  |  |  |  |
| ISCED 0-1 | 22.28 |  | 6.79 | 2.29 |
| ISCED 2 | 21.09 |  | 7.14 | 2.08 |
| ISCED 3 | 32.11 |  | 7.29 | 1.90 |
| ISCED 4 | 1.72 |  | 7.75 | 1.49 |
| ISCED 5-6 | 22.79 |  | 7.52 | 1.68 |
| Household total net income |  |  |  |  |
| Group 1 (< €6000) | 12.21 |  | 6.06 | 2.35 |
| Group 2 (€6000-18000) | 32.96 |  | 6.95 | 2.12 |
| Group 3 (€18000-30000) | 25.01 |  | 7.45 | 1.81 |
| Group 4 (€30000-60000) | 21.37 |  | 7.73 | 1.58 |
| Group 5 (> €60000) | 8.45 |  | 7.86 | 1.50 |
| Age group |  |  |  |  |
| 14-29 | 11.68 |  | 7.41 | 1.94 |
| 30-39 | 20.15 |  | 7.36 | 1.89 |
| 40-49 | 19.04 |  | 7.15 | 1.93 |
| 50-59 | 16.97 |  | 7.20 | 1.98 |
| 60-69 | 15.54 |  | 7.22 | 1.98 |
| 70 and above | 16.62 |  | 6.93 | 2.23 |
| Age | 49.90 (17.20) |  |  |  |
| Happiness | 7.21 (1.20) |  |  |  |
| Level 2: Country (*N*=12) |  |  |  |  |
| Gini | 0.31 (0.04) |  |  |  |
| GDP per capita annual growth (%) | 2.33 (1.97) |  |  |  |

Note: ISCED refers to “The International Standard Classification of Education”. Percentages for categorical variables and means for continuous variables are reported, and numbers in parentheses are standard deviations.

Table S5. Summary Statistics for All Measured Variables, Western European Countries, ESS 2004

| Variables | % or Mean |  | Happiness | |
| --- | --- | --- | --- | --- |
|  |  |  | Mean | Std. Dev. |
| Level-1: individual (*N*=11480) |  |  |  |  |
| Gender |  |  |  |  |
| Female | 50.93 |  | 7.23 | 1.92 |
| Male | 49.07 |  | 7.33 | 1.79 |
| Employment status |  |  |  |  |
| Employed | 52.71 |  | 7.47 | 1.63 |
| Unemployed | 5.95 |  | 6.38 | 2.10 |
| Retired | 27.40 |  | 7.07 | 2.04 |
| Home making | 13.94 |  | 7.34 | 2.01 |
| Marital status |  |  |  |  |
| Never married | 21.50 |  | 7.17 | 1.81 |
| Married | 59.27 |  | 7.54 | 1.72 |
| Widowed | 10.84 |  | 6.51 | 2.15 |
| Divorced | 6.56 |  | 6.86 | 1.99 |
| Separated | 1.83 |  | 6.33 | 2.23 |
| Living with child(ren) |  |  |  |  |
| No | 57.80 |  | 7.19 | 1.90 |
| Yes | 42.20 |  | 7.40 | 1.79 |
| Self-rated health |  |  |  |  |
| Unhealthy | 7.40 |  | 5.76 | 2.31 |
| Healthy | 92.60 |  | 7.40 | 1.76 |
| Education |  |  |  |  |
| ISCED 0-1 | 25.77 |  | 6.86 | 2.10 |
| ISCED 2 | 18.68 |  | 7.29 | 1.86 |
| ISCED 3 | 32.25 |  | 7.37 | 1.79 |
| ISCED 4 | 2.43 |  | 7.61 | 1.43 |
| ISCED 5-6 | 20.87 |  | 7.61 | 1.55 |
| Household total net income |  |  |  |  |
| Group 1 (< €6000) | 10.16 |  | 6.31 | 2.11 |
| Group 2 (€6000-18000) | 32.40 |  | 6.98 | 1.97 |
| Group 3 (€18000-30000) | 25.53 |  | 7.42 | 1.72 |
| Group 4 (€30000-60000) | 23.47 |  | 7.74 | 1.55 |
| Group 5 (> €60000) | 8.44 |  | 7.89 | 1.58 |
| Age group |  |  |  |  |
| 16-29 | 12.20 |  | 7.44 | 1.69 |
| 30-39 | 19.11 |  | 7.43 | 1.75 |
| 40-49 | 19.56 |  | 7.27 | 1.77 |
| 50-59 | 16.43 |  | 7.27 | 1.85 |
| 60-69 | 16.63 |  | 7.31 | 1.86 |
| 70 and above | 16.08 |  | 6.96 | 2.14 |
| Age | 50.34 (17.16) |  |  |  |
| Happiness | 7.28 (1.86) |  |  |  |
| Level 2: Country (*N*=11) |  |  |  |  |
| Gini | 0.30 (0.04) |  |  |  |
| GDP per capita annual growth (%) | 3.15 (1.19) |  |  |  |

Note: ISCED refers to “The International Standard Classification of Education”. Percentages for categorical variables and means for continuous variables are reported, and numbers in parentheses are standard deviations.

Table S6. Summary Statistics for All Measured Variables, Western European Countries, ESS 2006

| Variables | % or Mean |  | Happiness | |
| --- | --- | --- | --- | --- |
|  |  |  | Mean | Std. Dev. |
| Level-1: individual (*N*=11575) |  |  |  |  |
| Gender |  |  |  |  |
| Female | 50.37 |  | 7.39 | 1.84 |
| Male | 49.63 |  | 7.40 | 1.75 |
| Employment status |  |  |  |  |
| Employed | 58.73 |  | 7.49 | 1.67 |
| Unemployed | 5.45 |  | 6.50 | 2.11 |
| Retired | 25.78 |  | 7.28 | 1.91 |
| Home making | 10.04 |  | 7.58 | 1.87 |
| Marital status |  |  |  |  |
| Never married | 21.50 |  | 7.23 | 1.81 |
| Married | 58.08 |  | 7.64 | 1.66 |
| Widowed | 10.00 |  | 6.77 | 2.06 |
| Divorced | 8.64 |  | 7.03 | 1.92 |
| Separated | 1.77 |  | 6.64 | 2.13 |
| Living with child(ren) |  |  |  |  |
| No | 61.94 |  | 7.30 | 1.84 |
| Yes | 38.06 |  | 7.54 | 1.71 |
| Self-rated health |  |  |  |  |
| Unhealthy | 6.20 |  | 5.86 | 2.36 |
| Healthy | 93.80 |  | 7.49 | 1.70 |
| Education |  |  |  |  |
| ISCED 0-1 | 18.70 |  | 7.05 | 2.12 |
| ISCED 2 | 16.59 |  | 7.39 | 1.83 |
| ISCED 3 | 34.62 |  | 7.34 | 1.79 |
| ISCED 4 | 3.42 |  | 7.70 | 1.62 |
| ISCED 5-6 | 26.67 |  | 7.66 | 1.49 |
| Household total net income |  |  |  |  |
| Group 1 (< €6000) | 6.80 |  | 6.34 | 2.34 |
| Group 2 (€6000-18000) | 25.69 |  | 7.01 | 1.98 |
| Group 3 (€18000-30000) | 27.03 |  | 7.43 | 1.72 |
| Group 4 (€30000-60000) | 28.65 |  | 7.76 | 1.45 |
| Group 5 (> €60000) | 11.83 |  | 7.84 | 1.51 |
| Age group |  |  |  |  |
| 15-29 | 11.06 |  | 7.51 | 1.73 |
| 30-39 | 17.2 |  | 7.55 | 1.71 |
| 40-49 | 19.12 |  | 7.36 | 1.76 |
| 50-59 | 15.09 |  | 7.24 | 1.80 |
| 60-69 | 13.69 |  | 7.46 | 1.80 |
| 70 and above | 23.84 |  | 7.30 | 1.89 |
| Age | 50.42 (17.22) |  |  |  |
| Happiness | 7.39 (1.80) |  |  |  |
| Level 2: Country (*N*=10) |  |  |  |  |
| Gini | 0.30 (0.04) |  |  |  |
| GDP per capita annual growth (%) | 3.31 (1.04) |  |  |  |

Note: ISCED refers to “The International Standard Classification of Education”. Percentages for categorical variables and means for continuous variables are reported, and numbers in parentheses are standard deviations.

Table S7. Mixed-effects Ordered Logit Model of Happiness on Individual and Year Level Variables: Fixed Effects, GSS 1972-2010

|  | Fixed Effects | Coeff. | S.E. | T-ratio | *df* | *P*-value |
| --- | --- | --- | --- | --- | --- | --- |
| intercept, *β*_0_*_j_* | intercept, *γ*_00_ | -12.571 | 12.033 | -1.045 | 20 | 0.309 |
|  | Gini, *γ*_01_ | 26.023 | 10.177 | 2.557 | 20 | 0.019 |
|  | Gini squared, *γ*_02_ | -36.527 | 12.720 | -2.872 | 20 | 0.010 |
|  | GDP growth, *γ*_03_ | 0.015 | 0.006 | 2.461 | 20 | 0.023 |
|  | time, *γ*_04_ | 0.004 | 0.006 | 0.579 | 20 | 0.569 |
| slope of age, *β*_1_*_j_* | intercept, *γ*_10_ | 0.006 | 0.002 | 3.640 | 24 | 0.002 |
| slope of years of schooling, *β*_2_*_j_* | intercept, *γ*_20_ | 0.031 | 0.005 | 6.513 | 24 | 0.000 |
| slope of male, *β*_3_*_j_* | intercept, *γ*_30_ | -0.197 | 0.029 | -6.777 | 24 | 0.000 |
| slope of part-time job, *β*_4_*_j_* | intercept, *γ*_40_ | -0.031 | 0.027 | -1.153 | 24 | 0.261 |
| slope of temporarily unemployed, *β*_5_*_j_* | intercept, *γ*_50_ | -0.208 | 0.075 | -2.768 | 24 | 0.011 |
| slope of unemployed, *β*_6_*_j_* | intercept, *γ*_60_ | -0.647 | 0.075 | -8.642 | 24 | 0.000 |
| slope of retired, *β*_7_*_j_* | intercept, *γ*_70_ | 0.289 | 0.041 | 7.030 | 24 | 0.000 |
| slope of house-working, *β*_8_*_j_* | intercept, *γ*_80_ | 0.054 | 0.032 | 1.666 | 24 | 0.108 |
| slope of married, *β*_9_*_j_* | intercept, *γ*_90_ | 0.736 | 0.063 | 11.704 | 24 | 0.000 |
| slope of widowed, *β*_10_*_j_* | intercept, *γ*_100_ | -0.184 | 0.067 | -2.754 | 24 | 0.011 |
| slope of divorced, *β*_11_*_j_* | intercept, *γ*_110_ | -0.197 | 0.057 | -3.464 | 24 | 0.002 |
| slope of separated, *β*_12_*_j_* | intercept, *γ*_120_ | -0.426 | 0.072 | -5.910 | 24 | 0.000 |
| slope of black, *β*_13_*_j_* | intercept, *γ*_130_ | -0.346 | 0.055 | -6.270 | 24 | 0.000 |
| slope of living with child, *β*_14_*_j_* | intercept, *γ*_140_ | -0.160 | 0.027 | -5.945 | 24 | 0.000 |
| slope of healthy, *β*_15_*_j_* | intercept, *γ*_150_ | 1.101 | 0.051 | 21.596 | 24 | 0.000 |
| slope of logarithm of income, *β*_16_*_j_* | intercept, *γ*_160_ | -0.284 | 0.259 | -1.096 | 23 | 0.285 |
|  | Gini Coefficient, *γ*_161_ | 1.890 | 0.632 | 2.991 | 23 | 0.007 |
| slope of household size, | Gini Coefficient, *γ*_161_ | -0.025 | 0.011 | -2.403 | 24 | 0.024 |
| Threshold, *δ*_2_ |  | 2.990 | 0.037 | 79.805 | 31247 | 0.000 |

Table S8. Mixed-effects Ordered Logit Model of Happiness on Individual and year level variables: Random Effects, GSS 1972-2010

|  | Variance Component | *df* | Chi-square | P-value |
| --- | --- | --- | --- | --- |
| intercept, *μ*_0_*_j_* | 0.010 | 20 | 89.616 | 0.000 |
| slope of age, *μ*_1_*_j_* | 0.000 | 24 | 70.414 | 0.000 |
| slope of years of schooling, *μ*_2_*_j_* | 0.000 | 24 | 33.236 | 0.099 |
| slope of male, *μ*_3_*_j_* | 0.013 | 24 | 33.444 | 0.095 |
| slope of part-time job, *μ*_4_*_j_* | 0.004 | 25 | 12.423 | >.500 |
| slope of temporarily unemployed, *μ*_5_*_j_* | 0.061 | 25 | 24.301 | >.500 |
| slope of unemployed, *μ*_6_*_j_* | 0.070 | 25 | 30.749 | 0.197 |
| slope of retired, *μ*_7_*_j_* | 0.017 | 25 | 21.351 | >.500 |
| slope of house-keeping, *μ*_8_*_j_* | 0.012 | 25 | 17.058 | >.500 |
| slope of married, *μ*_9_*_j_* | 0.075 | 24 | 60.276 | 0.000 |
| slope of widowed, *μ*_10_*_j_* | 0.065 | 24 | 32.459 | 0.116 |
| slope of divorced, *μ*_11_*_j_* | 0.042 | 24 | 34.147 | 0.082 |
| slope of separated, *μ*_12_*_j_* | 0.050 | 24 | 23.229 | >.500 |
| slope of black, *μ*_13_*_j_* | 0.053 | 25 | 62.480 | 0.000 |
| slope of living with child, *μ*_14_*_j_* | 0.005 | 25 | 18.198 | >.500 |
| slope of healthy, *μ*_15_*_j_* | 0.022 | 25 | 19.958 | >.500 |
| slope of logarithm of income, *μ*_16_*_j_* | 0.025 | 23 | 35.528 | 0.046 |
| slope of household size | 0.037 | 0.001 | 25 | 31.492 |

Table S9. Mixed-Effects Linear Model of Happiness on Individual and Country Level Variables: Fixed Effects, ESS 2002

|  | Fixed Effects | Coeff. | S.E. | T-ratio | *df* | P-value |
| --- | --- | --- | --- | --- | --- | --- |
| intercept, *β*_0_*_j_* | intercept, *γ*_00_ | 0.301 | 2.270 | 0.13 | 7 | 0.899 |
|  | Gini, *γ*_01_ | 51.326 | 14.980 | 3.43 | 7 | 0.013 |
|  | Gini squared, *γ*_02_ | -90.728 | 24.649 | -3.68 | 7 | 0.010 |
|  | GDP growth, *γ*_03_ | -0.021 | 0.034 | -0.62 | 7 | 0.554 |
| slope of healthy, *β*_1_*_j_* | intercept, *γ*_10_ | 1.251 | 0.105 | 11.94 | 10 | 0.000 |
| slope of household size, *β*_2_*_j_* | intercept, *γ*_20_ | 0.043 | 0.026 | 1.68 | 10 | 0.123 |
| slope of male, *β*_3_*_j_* | intercept, *γ*_30_ | -0.123 | 0.052 | -2.37 | 10 | 0.039 |
| slope of age, *β*_4_*_j_* | intercept, *γ*_40_ | -0.003 | 0.004 | -0.78 | 10 | 0.453 |
| slope of unemployed, *β*_5_*_j_* | intercept, *γ*_50_ | -0.571 | 0.193 | -2.96 | 10 | 0.015 |
| slope of retired, *β*_6_*_j_* | intercept, *γ*_60_ | 0.213 | 0.075 | 2.86 | 10 | 0.018 |
| slope of house-working, *β*_7_*_j_* | intercept, *γ*_70_ | -0.030 | 0.075 | -0.40 | 10 | 0.696 |
| slope of living with child, *β*_8_*_j_* | intercept, *γ*_80_ | -0.183 | 0.070 | -2.63 | 10 | 0.026 |
| slope of separated, *β*_9_*_j_* | intercept, *γ*_90_ | -1.099 | 0.170 | -6.46 | 10 | 0.000 |
| slope of divorced, *β*_10_*_j_* | intercept, *γ*_100_ | -0.689 | 0.133 | -5.19 | 10 | 0.000 |
| slope of widowed, *β*_11_*_j_* | intercept, *γ*_110_ | -0.608 | 0.107 | -5.69 | 10 | 0.000 |
| slope of single, *β*_12_*_j_* | intercept, *γ*_120_ | -0.396 | 0.079 | -5.03 | 10 | 0.000 |
| slope of less than lower secondary education, *β*_13_*_j_* | intercept, *γ*_130_ | -0.107 | 0.085 | -1.26 | 10 | 0.238 |
| slope of upper secondary education, *β*_14_*_j_* | intercept, *γ*_140_ | -0.084 | 0.076 | -1.11 | 10 | 0.293 |
| slope of post-secondary non-tertiary education, *β*_15_*_j_* | intercept, *γ*_150_ | 0.172 | 0.135 | 1.27 | 10 | 0.233 |
| slope of tertiary education, *β*_16_*_j_* | intercept, *γ*_160_ | 0.100 | 0.058 | 1.72 | 10 | 0.115 |
| slope of income group 1, *β*_17_*_j_* | intercept, *γ*_170_ | -0.489 | 0.166 | -2.94 | 10 | 0.015 |
| slope of income group 2, *β*_18_*_j_* | intercept, *γ*_180_ | -0.209 | 0.081 | -2.57 | 10 | 0.028 |
| slope of income group 4, *β*_19_*_j_* | intercept, *γ*_190_ | 0.084 | 0.100 | 0.84 | 10 | 0.421 |
| slope of income group 5, *β*_20_*_j_* | intercept, *γ*_200_ | 0.154 | 0.079 | 1.94 | 10 | 0.080 |

Note: Belgium, Switzerland, Germany, Spain, United Kingdom, Greece, Ireland, Italy, Luxembourg, Netherlands, Portugal and Slovenia are included.

Table S10. Mixed-Effects Linear Model of Happiness on Individual and Country Level Variables: Random Effects, ESS 2002

|  | Variance Component | *df* | Chi-square | P-value |
| --- | --- | --- | --- | --- |
| intercept，*μ*_0_*_j_* | 0.148 | 0 | 91.515 | >.500 |
| slope of healthy, *μ*_1_*_j_* | 0.067 | 3 | 4.591 | 0.203 |
| slope of household size, *μ*_2_*_j_* | 0.003 | 3 | 4.785 | 0.187 |
| slope of male, *μ*_3_*_j_* | 0.013 | 3 | 2.265 | >.500 |
| slope of age, *μ*_4_*_j_* | 0.000 | 3 | 16.430 | 0.001 |
| slope of unemployed, *μ*_5_*_j_* | 0.321 | 3 | 34.817 | 0.000 |
| slope of retired, *μ*_6_*_j_* | 0.020 | 3 | 1.481 | >.500 |
| slope of house-working, *μ*_7_*_j_* | 0.024 | 3 | 3.764 | 0.287 |
| slope of living with child, *μ*_8_*_j_* | 0.024 | 3 | 4.656 | 0.197 |
| slope of separated, *μ*_9_*_j_* | 0.117 | 3 | 2.219 | >.500 |
| slope of divorced, *μ*_10_*_j_* | 0.126 | 3 | 1.817 | >.500 |
| slope of widowed, *μ*_11_*_j_* | 0.077 | 3 | 5.430 | 0.141 |
| slope of single, *μ*_12_*_j_* | 0.034 | 3 | 3.634 | 0.303 |
| slope of less than lower secondary education, *μ*_13_*_j_* | 0.038 | 3 | 0.993 | >.500 |
| slope of upper secondary education, *μ*_14_*_j_* | 0.033 | 3 | 6.314 | 0.096 |
| slope of post-secondary non-tertiary education, *μ*_15_*_j_* | 0.013 | 3 | 0.830 | >.500 |
| slope of tertiary education, *μ*_16_*_j_* | 0.009 | 3 | 1.636 | >.500 |
| slope of income group 1, *μ*_17_*_j_* | 0.233 | 3 | 10.361 | 0.016 |
| slope of income group 2, *μ*_1_*_j_* | 0.044 | 3 | 4.210 | 0.238 |
| slope of income group 4, *μ*_19_*_j_* | 0.078 | 3 | 3.824 | 0.280 |
| slope of income group 5, *μ*_20_*_j_* | 0.014 | 3 | 0.876 | >.500 |
| Individual-level, *e_ij_* | 3.139 |  |  |  |

Note: Belgium, Switzerland, Germany, Spain, United Kingdom, Greece, Ireland, Italy, Luxembourg, Netherlands, Portugal and Slovenia are included.

Table S11. Mixed-effects Linear Model of Happiness on Individual and Country Level Variables: Fixed Effects, ESS 2004

|  | Fixed Effects | Coeff. | S.E. | T-ratio | *df* | P-value |
| --- | --- | --- | --- | --- | --- | --- |
| intercept, *β*_0_*_j_* | intercept, *γ*_00_ | -4.345 | 3.358 | -1.294 | 7 | 0.237 |
|  | Gini, *γ*_01_ | 76.770 | 22.399 | 3.427 | 7 | 0.013 |
|  | Gini squared, *γ*_02_ | -127.393 | 36.274 | -3.512 | 7 | 0.012 |
|  | GDP growth, *γ*_03_ | 0.101 | 0.047 | 2.141 | 7 | 0.069 |
| slope of healthy, *β*_1_*_j_* | intercept, *γ*_10_ | 0.721 | 0.094 | 7.641 | 10 | 0.000 |
| slope of household size, *β*_2_*_j_* | intercept, *γ*_20_ | 0.050 | 0.026 | 1.907 | 10 | 0.085 |
| slope of male, *β*_3_*_j_* | intercept, *γ*_30_ | -0.102 | 0.046 | -2.244 | 10 | 0.049 |
| slope of age, *β*_4_*_j_* | intercept, *γ*_40_ | -0.002 | 0.004 | -0.655 | 10 | 0.527 |
| slope of unemployed, *β*_5_*_j_* | intercept, *γ*_50_ | -0.648 | 0.136 | -4.769 | 10 | 0.001 |
| slope of retired, *β*_6_*_j_* | intercept, *γ*_60_ | 0.223 | 0.087 | 2.560 | 10 | 0.029 |
| slope of house-working, *β*_7_*_j_* | intercept, *γ*_70_ | 0.046 | 0.085 | 0.546 | 10 | 0.596 |
| slope of living with child, *β*_8_*_j_* | intercept, *γ*_80_ | -0.161 | 0.051 | -3.127 | 10 | 0.011 |
| slope of separated, *β*_9_*_j_* | intercept, *γ*_90_ | -1.157 | 0.198 | -5.841 | 10 | 0.000 |
| slope of divorced, *β*_10_*_j_* | intercept, *γ*_100_ | -0.577 | 0.106 | -5.431 | 10 | 0.000 |
| slope of widowed, *β*_11_*_j_* | intercept, *γ*_110_ | -0.636 | 0.118 | -5.398 | 10 | 0.000 |
| slope of single, *β*_12_*_j_* | intercept, *γ*_120_ | -0.426 | 0.088 | -4.855 | 10 | 0.000 |
| slope of less than lower secondary education, *β*_13_*_j_* | intercept, *γ*_130_ | -0.067 | 0.071 | -0.944 | 10 | 0.368 |
| slope of upper secondary education, *β*_14_*_j_* | intercept, *γ*_140_ | -0.051 | 0.067 | -0.764 | 10 | 0.462 |
| slope of post-secondary non-tertiary education, *β*_15_*_j_* | intercept, *γ*_150_ | 0.148 | 0.134 | 1.107 | 10 | 0.295 |
| slope of tertiary education, *β*_16_*_j_* | intercept, *γ*_160_ | 0.060 | 0.059 | 1.008 | 10 | 0.338 |
| slope of income group 2, *β*_17_*_j_* | intercept, *γ*_170_ | 0.037 | 0.134 | 0.277 | 10 | 0.787 |
| slope of income group 3, *β*_18_*_j_* | intercept, *γ*_180_ | 0.194 | 0.144 | 1.342 | 10 | 0.209 |
| slope of income group 4, *β*_19_*_j_* | intercept, *γ*_190_ | 0.302 | 0.129 | 2.338 | 10 | 0.041 |
| slope of income group 5, *β*_20_*_j_* | intercept, *γ*_200_ | 0.383 | 0.138 | 2.777 | 10 | 0.020 |

Note: Austria, Belgium, Germany, Spain, United Kingdom, Greece, Ireland, Luxembourg, Netherlands, Portugal, and Slovenia are included.

Table S12. Mixed-effects Linear Model of Happiness on Individual and Country Level Variables: Random Effects, ESS 2004

| Random Effects | Variance Component | *df* | Chi-square | P-value |
| --- | --- | --- | --- | --- |
| intercept，*μ*_0_*_j_* | 0.148 | 1 | 92.123 | 0.000 |
| slope of healthy, *μ*_1_*_j_* | 0.082 | 4 | 44.261 | 0.000 |
| slope of household size, *μ*_2_*_j_* | 0.004 | 4 | 5.773 | 0.216 |
| slope of male, *μ*_3_*_j_* | 0.009 | 4 | 1.097 | >.500 |
| slope of age, *μ*_4_*_j_* | 0.000 | 4 | 6.409 | 0.169 |
| slope of unemployed, *μ*_5_*_j_* | 0.143 | 4 | 18.994 | 0.001 |
| slope of retired, *μ*_6_*_j_* | 0.046 | 4 | 7.174 | 0.126 |
| slope of house-working, *μ*_7_*_j_* | 0.044 | 4 | 8.667 | 0.069 |
| slope of living with child, *μ*_8_*_j_* | 0.005 | 4 | 2.298 | >.500 |
| slope of separated, *μ*_9_*_j_* | 0.257 | 4 | 5.061 | 0.280 |
| slope of divorced, *μ*_10_*_j_* | 0.069 | 4 | 5.276 | 0.259 |
| slope of widowed, *μ*_11_*_j_* | 0.110 | 4 | 9.305 | 0.053 |
| slope of single, *μ*_12_*_j_* | 0.055 | 4 | 13.573 | 0.009 |
| slope of less than lower secondary education, *μ*_13_*_j_* | 0.023 | 4 | 1.484 | >.500 |
| slope of upper secondary education, *μ*_14_*_j_* | 0.022 | 4 | 1.945 | >.500 |
| slope of post-secondary non-tertiary education, *μ*_15_*_j_* | 0.064 | 4 | 6.348 | 0.173 |
| slope of tertiary education, *μ*_16_*_j_* | 0.014 | 4 | 3.607 | >.500 |
| slope of income group 2, *μ*_17_*_j_* | 0.146 | 4 | 22.088 | 0.000 |
| slope of income group 3, *μ*_1_*_j_* | 0.169 | 4 | 16.859 | 0.002 |
| slope of income group 4, *μ*_19_*_j_* | 0.113 | 4 | 8.837 | 0.064 |
| slope of income group 5, *μ*_20_*_j_* | 0.112 | 4 | 6.065 | 0.193 |
| Individual-level, *e_ij_* | 2.802 |  |  |  |

Note: Austria, Belgium, Germany, Spain, United Kingdom, Greece, Ireland, Luxembourg, Netherlands, Portugal, and Slovenia are included.

Table S13. Mixed-effects Linear Model of Happiness on Individual and Country Level Variables: Fixed Effects, ESS 2006

|  | Fixed Effects | Coeff. | S.E. | T-ratio | *df* | P-value |
| --- | --- | --- | --- | --- | --- | --- |
| intercept, *β*_0_*_j_* | intercept, *γ*_00_ | -19.921 | 3.198 | -6.229 | 6 | 0.000 |
|  | Gini, *γ*_01_ | 180.797 | 21.567 | 8.383 | 6 | 0.000 |
|  | Gini squared, *γ*_02_ | -295.310 | 34.947 | -8.450 | 6 | 0.000 |
|  | GDP growth, *γ*_03_ | 0.027 | 0.058 | 0.467 | 6 | 0.656 |
| slope of healthy, *β*_1_*_j_* | intercept, *γ*_10_ | 1.247 | 0.161 | 7.765 | 9 | 0.000 |
| slope of household size, *β*_2_*_j_* | intercept, *γ*_20_ | 0.087 | 0.026 | 3.363 | 9 | 0.009 |
| slope of male, *β*_3_*_j_* | intercept, *γ*_30_ | -0.154 | 0.045 | -3.424 | 9 | 0.009 |
| slope of age, *β*_4_*_j_* | intercept, *γ*_40_ | -0.002 | 0.002 | -0.786 | 9 | 0.452 |
| slope of unemployed, *β*_5_*_j_* | intercept, *γ*_50_ | -0.458 | 0.143 | -3.199 | 9 | 0.012 |
| slope of retired, *β*_6_*_j_* | intercept, *γ*_60_ | 0.299 | 0.072 | 4.151 | 9 | 0.003 |
| slope of house-working, *β*_7_*_j_* | intercept, *γ*_70_ | 0.123 | 0.089 | 1.372 | 9 | 0.204 |
| slope of living with child, *β*_8_*_j_* | intercept, *γ*_80_ | -0.136 | 0.057 | -2.375 | 9 | 0.042 |
| slope of separated, *β*_9_*_j_* | intercept, *γ*_90_ | -0.742 | 0.169 | -4.382 | 9 | 0.002 |
| slope of divorced, *β*_10_*_j_* | intercept, *γ*_100_ | -0.362 | 0.100 | -3.618 | 9 | 0.006 |
| slope of widowed, *β*_11_*_j_* | intercept, *γ*_110_ | -0.559 | 0.103 | -5.415 | 9 | 0.000 |
| slope of single, *β*_12_*_j_* | intercept, *γ*_120_ | -0.296 | 0.083 | -3.586 | 9 | 0.007 |
| slope of less than lower secondary education, *β*_13_*_j_* | intercept, *γ*_130_ | -0.017 | 0.129 | -0.130 | 9 | 0.900 |
| slope of upper secondary education, *β*_14_*_j_* | intercept, *γ*_140_ | -0.040 | 0.079 | -0.506 | 9 | 0.625 |
| slope of post-secondary non-tertiary education, *β*_15_*_j_* | intercept, *γ*_150_ | 0.183 | 0.097 | 1.893 | 9 | 0.090 |
| slope of tertiary education, *β*_16_*_j_* | intercept, *γ*_160_ | 0.137 | 0.053 | 2.588 | 9 | 0.030 |
| slope of income group 2, *β*_17_*_j_* | intercept, *γ*_170_ | 0.141 | 0.152 | 0.928 | 9 | 0.378 |
| slope of income group 3, *β*_18_*_j_* | intercept, *γ*_180_ | 0.323 | 0.161 | 2.003 | 9 | 0.076 |
| slope of income group 4, *β*_19_*_j_* | intercept, *γ*_190_ | 0.486 | 0.205 | 2.370 | 9 | 0.042 |
| slope of income group 5, *β*_20_*_j_* | intercept, *γ*_200_ | 0.347 | 0.225 | 1.544 | 9 | 0.157 |

Note: Austria, Belgium, Switzerland, Germany, Spain, France, United Kingdom, Ireland, Netherlands, and Portugal are included.

Table S14. Mixed-effects Linear Model of Happiness on Individual and Country Level Variables: Random Effects, ESS 2006

|  | Variance Component | *df* | Chi-square | P-value |
| --- | --- | --- | --- | --- |
| intercept，*μ*_0_*_j_* | 0.089 | 1 | 267.864 | 0.000 |
| slope of healthy, *μ*_1_*_j_* | 0.204 | 4 | 37.857 | 0.000 |
| slope of household size, *μ*_2_*_j_* | 0.003 | 4 | 7.807 | 0.098 |
| slope of male, *μ*_3_*_j_* | 0.009 | 4 | 6.339 | 0.174 |
| slope of age, *μ*_4_*_j_* | 0.000 | 4 | 6.966 | 0.136 |
| slope of unemployed, *μ*_5_*_j_* | 0.152 | 4 | 13.555 | 0.009 |
| slope of retired, *μ*_6_*_j_* | 0.020 | 4 | 2.948 | >.500 |
| slope of house-working, *μ*_7_*_j_* | 0.046 | 4 | 6.114 | 0.189 |
| slope of living with child, *μ*_8_*_j_* | 0.010 | 4 | 2.051 | >.500 |
| slope of separated, *μ*_9_*_j_* | 0.142 | 4 | 8.582 | 0.071 |
| slope of divorced, *μ*_10_*_j_* | 0.064 | 4 | 14.494 | 0.006 |
| slope of widowed, *μ*_11_*_j_* | 0.069 | 4 | 13.392 | 0.010 |
| slope of single, *μ*_12_*_j_* | 0.045 | 4 | 14.884 | 0.005 |
| slope of less than lower secondary education, *μ*_13_*_j_* | 0.129 | 4 | 19.490 | 0.001 |
| slope of upper secondary education, *μ*_14_*_j_* | 0.040 | 4 | 11.573 | 0.021 |
| slope of post-secondary non-tertiary education, *μ*_15_*_j_* | 0.015 | 4 | 2.203 | >.500 |
| slope of tertiary education, *μ*_16_*_j_* | 0.010 | 4 | 2.847 | >.500 |
| slope of income group 2, *μ*_17_*_j_* | 0.177 | 4 | 15.572 | 0.004 |
| slope of income group 3, *μ*_1_*_j_* | 0.201 | 4 | 19.298 | 0.001 |
| slope of income group 4, *μ*_19_*_j_* | 0.358 | 4 | 36.371 | 0.000 |
| slope of income group 5, *μ*_20_*_j_* | 0.421 | 4 | 42.700 | 0.000 |
| Individual-level, *e_ij_* | 2.639 |  |  |  |

Note: Austria, Belgium, Switzerland, Germany, Spain, France, United Kingdom, Ireland, Netherlands, and Portugal are included.

| Table S15：Regression of Happiness on Gini coefficient | | | | | |
| --- | --- | --- | --- | --- | --- |
|  |  | Coeff. | S.E. | T | P-value |
| 25 Per | Constant | 2.598 | 0.063 | 41.070 | <0.001 |
|  | gini | 0.742 | 0.309 | 2.400 | =0.017 |
|  | gini2 | -6.098 | 0.355 | -17.180 | <0.001 |
| 50 Per | Constant | 2.318 | 0.049 | 47.156 | <0.001 |
|  | gini | 3.364 | 0.240 | 14.010 | <0.001 |
|  | gini2 | -5.864 | 0.276 | -21.263 | <0.001 |
| 75 Per | Constant | 2.409 | 0.045 | 53.949 | <0.001 |
|  | gini | 3.397 | 0.218 | 15.574 | <0.001 |
|  | gini2 | -3.658 | 0.251 | -14.602 | <0.001 |

Table S16. Mixed-effects Ordered Logit Model of Happiness on Individual and Year Level Variables: Fixed Effects, GSS 1972-2010 (including all races)

|  | Fixed Effects | Coeff. | S.E. | T-ratio | *df* | *P*-value |
| --- | --- | --- | --- | --- | --- | --- |
| intercept, *β*_0_*_j_* | intercept, *γ*_00_ | -12.055 | 1.795 | -6.716 | 22 | <0.001 |
|  | Gini, *γ*_01_ | 54.470 | 8.625 | 6.315 | 22 | <0.001 |
|  | Gini squared, *γ*_02_ | -68.846 | 10.396 | -6.622 | 22 | <0.001 |
| slope of age, *β*_1_*_j_* | intercept, *γ*_10_ | 0.009 | 0.002 | 5.936 | 24 | <0.001 |
| slope of years of schooling, *β*_2_*_j_* | intercept, *γ*_20_ | 0.008 | 0.005 | 1.774 | 24 | 0.088 |
| slope of female, *β*_3_*_j_* | intercept, *γ*_30_ | 0.195 | 0.034 | 5.803 | 24 | <0.001 |
| slope of working fulltime, *β*_4_*_j_* | intercept, *γ*_40_ | -0.110 | 0.104 | -1.053 | 24 | 0.303 |
| slope of part-time job, *β*_4_*_j_* | intercept, *γ*_40_ | -0.148 | 0.104 | -1.427 | 24 | 0.166 |
| slope of temp not working, *β*_5_*_j_* | intercept, *γ*_50_ | -0.264 | 0.135 | -1.958 | 24 | 0.061 |
| slope of unempl, laid off, *β*_6_*_j_* | intercept, *γ*_60_ | -0.709 | 0.117 | -6.051 | 24 | <0.001 |
| slope of retired, *β*_7_*_j_* | intercept, *γ*_70_ | 0.224 | 0.109 | 2.051 | 24 | 0.051 |
| slope ofstudent, *β*_8_*_j_* | intercept, *γ*_80_ | 0.133 | 0.134 | 0.991 | 24 | 0.332 |
| slope of keeping house, *β*_9_*_j_* | intercept, *γ*_90_ | 0.000 | 0.097 | -0.003 | 24 | 0.998 |
| slope of married, *β*_10_*_j_* | intercept, *γ*_100_ | 0.792 | 0.052 | 15.337 | 24 | <0.001 |
| slope of widowed, *β*_11_*_j_* | intercept, *γ*_110_ | -0.239 | 0.069 | -3.455 | 24 | 0.002 |
| slope of divorced, *β*_12_*_j_* | intercept, *γ*_120_ | -0.208 | 0.061 | -3.424 | 24 | 0.003 |
| slope of separated, *β*_13_*_j_* | intercept, *γ*_130_ | -0.375 | 0.069 | -5.429 | 24 | <0.001 |
| slope of black, *β*_14_*_j_* | intercept, *γ*_140_ | -0.313 | 0.058 | -5.413 | 24 | <0.001 |
| slope of other race, *β*_15_*_j_* | intercept, *γ*_150_ | -0.093 | 0.060 | -1.542 | 24 | 0.136 |
| slope of living with child, *β*_16_*_j_* | intercept, *γ*_160_ | -0.143 | 0.026 | -5.412 | 24 | <0.001 |
| slope of healthy, *β*_17_*_j_* | intercept, *γ*_170_ | 0.643 | 0.019 | 33.715 | 24 | <0.001 |
| slope of logarithm of income, *β*_18_*_j_* | intercept, *γ*_180_ | 0.263 | 0.030 | 8.777 | 24 | <0.001 |
| Threshold, *δ*_2_ |  | 3.069 | 0.038 | 79.939 | 34128 | <0.001 |

Table S17. Mixed-effects Ordered Logit Model of Happiness on Individual and Year Level Variables: Fixed Effects, GSS 1972-2010 (including all races)

|  | Fixed Effects | Coeff. | S.E. | T-ratio | *df* | *P*-value |
| --- | --- | --- | --- | --- | --- | --- |
| intercept, *β*_0_*_j_* | intercept, *γ*_00_ | -3.737 | 3.346 | -1.117 | 22 | 0.277 |
|  | Gini, *γ*_01_ | 16.788 | 16.703 | 1.005 | 22 | 0.326 |
|  | Gini squared, *γ*_02_ | -23.250 | 20.728 | -1.122 | 22 | 0.275 |
| Threshold, *δ*_2_ |  | 2.703 | 0.017 | 155.388 | 34147 | 0.000 |

Table S18. Mixed-effects Ordered Logit Model of Happiness on Individual and Year Level Variables: Fixed Effects, GSS 1972-2010

|  | Fixed Effects | Coeff. | S.E. | T-ratio | *df* | *P*-value |
| --- | --- | --- | --- | --- | --- | --- |
| intercept, *β*_0_*_j_* | intercept, *γ*_00_ | -6.744 | 2.698 | -2.499 | 22 | 0.021 |
|  | Gini, *γ*_01_ | 30.938 | 13.468 | 2.297 | 22 | 0.032 |
|  | Gini squared, *γ*_02_ | -40.935 | 16.669 | -2.456 | 22 | 0.023 |
| slope of age, *β*_1_*_j_* | intercept, *γ*_10_ | 0.011 | 0.001 | 9.982 | 24 | <0.001 |
| slope of years of schooling, *β*_2_*_j_* | intercept, *γ*_20_ | 0.010 | 0.005 | 2.268 | 24 | 0.033 |
| slope of female, *β*_3_*_j_* | intercept, *γ*_30_ | 0.119 | 0.032 | 3.761 | 24 | 0.001 |
| slope of black, *β*_4_*_j_* | intercept, *γ*_140_ | -0.482 | 0.052 | -9.332 | 24 | <0.001 |
| slope of other race, *β*_5_*_j_* | intercept, *γ*_150_ | -0.068 | 0.057 | -1.191 | 24 | 0.246 |
| slope of healthy, *β*_6_*_j_* | intercept, *γ*_160_ | 0.652 | 0.020 | 32.299 | 24 | <0.001 |
| slope of logarithm of income, *β*_7_*_j_* | intercept, *γ*_170_ | 0.305 | 0.027 | 11.202 | 24 | <0.001 |
| Threshold, *δ*_2_ |  | 2.930 | 0.036 | 80.328 | 34140 | <0.001 |

Table S19. Mixed-Effects Linear Model of Happiness on Individual and Country Level Variables: Fixed Effects, ESS 2002

|  | Fixed Effects | Coeff. | S.E. | T-ratio | *df* | P-value |
| --- | --- | --- | --- | --- | --- | --- |
| intercept, *β*_0_*_j_* | intercept, *γ*_00_ | -5.815 | 2.900 | -2.005 | 9 | 0.075 |
|  | Gini, *γ*_01_ | 95.612 | 20.693 | 4.620 | 9 | 0.001 |
|  | Gini squared, *γ*_02_ | -168.366 | 36.120 | -4.661 | 9 | 0.001 |

Table S20. Mixed-Effects Linear Model of Happiness on Individual and Country Level Variables: Fixed Effects, ESS 2004

|  | Fixed Effects | Coeff. | S.E. | T-ratio | *df* | P-value |
| --- | --- | --- | --- | --- | --- | --- |
| intercept, *β*_0_*_j_* | intercept, *γ*_00_ | -4.349 | 6.649 | -0.654 | 8 | 0.531 |
|  | Gini, *γ*_01_ | 82.678 | 43.169 | 1.915 | 8 | 0.091 |
|  | Gini squared, *γ*_02_ | -143.355 | 67.810 | -2.114 | 8 | 0.067 |

Table S21. Mixed-Effects Linear Model of Happiness on Individual and Country Level Variables: Fixed Effects, ESS 2006

|  | Fixed Effects | Coeff. | S.E. | T-ratio | *df* | P-value |
| --- | --- | --- | --- | --- | --- | --- |
| intercept, *β*_0_*_j_* | intercept, *γ*_00_ | -14.594 | 3.492 | -4.179 | 7 | 0.005 |
|  | Gini, *γ*_01_ | 147.221 | 21.594 | 6.818 | 7 | 0.000 |
|  | Gini squared, *γ*_02_ | -242.446 | 32.821 | -7.387 | 7 | 0.000 |

Table S22. Mixed-Effects Linear Model of Happiness on Individual and Country Level Variables: Fixed Effects, ESS 2002

|  | Fixed Effects | Coeff. | S.E. | T-ratio | *df* | P-value |
| --- | --- | --- | --- | --- | --- | --- |
| intercept, *β*_0_*_j_* | intercept, *γ*_00_ | 8.142 | 4.762 | 1.710 | 12 | 0.113 |
|  | Gini, *γ*_01_ | 2.907 | 31.163 | 0.093 | 12 | 0.928 |
|  | Gini squared, *γ*_02_ | -14.848 | 49.749 | -0.298 | 12 | 0.770 |

Note: Add the data of Denmark, Finland, Norway and Sweden.

Table S23. Mixed-Effects Linear Model of Happiness on Individual and Country Level Variables: Fixed Effects, ESS 2004

|  | Fixed Effects | Coeff. | S.E. | T-ratio | *df* | P-value |
| --- | --- | --- | --- | --- | --- | --- |
| intercept, *β*_0_*_j_* | intercept, *γ*_00_ | 10.563 | 4.557 | 2.318 | 12 | 0.039 |
|  | Gini, *γ*_01_ | -10.699 | 30.359 | -0.352 | 12 | 0.730 |
|  | Gini squared, *γ*_02_ | 0.612 | 48.988 | 0.012 | 12 | 0.990 |

Note: Add the data of Denmark, Iceland, Finland, Norway and Sweden.

Table S24. Mixed-Effects Linear Model of Happiness on Individual and Country Level Variables: Fixed Effects, ESS 2006

|  | Fixed Effects | Coeff. | S.E. | T-ratio | *df* | P-value |
| --- | --- | --- | --- | --- | --- | --- |
| intercept, *β*_0_*_j_* | intercept, *γ*_00_ | 20.055 | 8.946 | 2.240 | 10 | 0.049 |
|  | Gini, *γ*_01_ | -90.560 | 64.073 | -1.413 | 10 | 0.188 |
|  | Gini squared, *γ*_02_ | 163.00 | 113.425 | 1.437 | 10 | 0.181 |

Figure 1. Mean of Household Annual Income in U.S., 1970-2010 (Unit:$)

Figure 2. Gini Coefficient in U.S., 1972-2010

Figure 3. Mean of Happiness Rating in U.S., 1972-2010

### Model Specifications in Study One

We denoted the respondent’s happiness level as *R* and used cumulative probability to reflect the ordinal nature of the response:

（1）

（2）


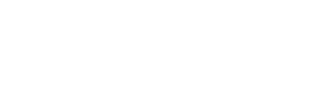


（3）

where

（4）


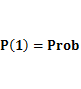


（5）

Note that (3) is redundant because = 1 – (+ ).

At level 1, the key independent variable was household annual income. We performed a logarithmic transformation on household income (Kahneman & Deaton, 2010). Demographic variables such as age, gender (male = 1, female = 0), year of schooling, marriage status, household size, occupation and race were included as control variables. Marriage status was transformed into four dummy variables representing ‘married’, ‘widowed’, ‘divorced’ and ‘separated’, respectively, with ‘unmarried’ as the reference. Race was also dummy-coded with Whites as the reference. After including log income as a predictor, level 1 equation becomes:

（6）


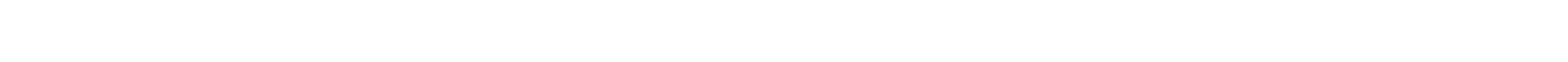


（7）


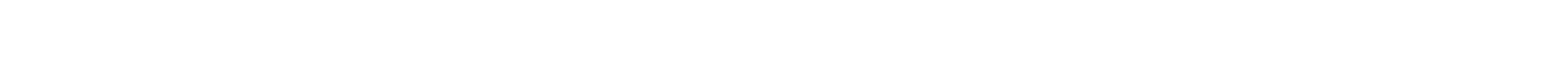

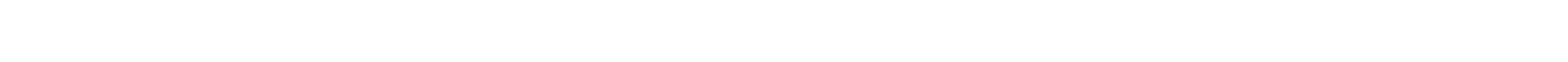


where *i* and *j* denotes individual respondent and survey year, respectively.

Equations 6 and 7 share the same regression coefficients. The only difference between them is the intercept. The intercept of equation 6 is$\beta_{0}\text{+}\delta_{1}$, while that of equation 7 is$\beta_{0}\text{+}\delta_{2}$. However, equations with different intercepts are difficult to interpret. Therefore, we separated the error term (*m* = 1，2) to yield the intercept$\beta_{0}$.


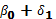


Let , the level 1 or individual level structural model becomes:


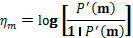


$\begin{matrix} h_{mj}=\beta_{0j}+\beta_{1j}*age_{ij}+\beta_{2j}*schyr_{ij}+\beta_{3j}*male_{ij}+\beta_{4j}*parttime_{ij} \\ +\beta_{5j}*tempunemp_{ij}+\beta_{6j}*unemp_{ij}+\beta_{7j}*retired_{ij}+\beta_{8j}*keephouse_{ij} \\ +\beta_{9j}*married_{ij}+\beta_{10j}*widowed_{ij}+\beta_{11j}*divorced_{ij}+\beta_{12j}*separated_{ij} \\ +b_{13j}*black_{ij}+\beta_{14j}*child_{ij}+\beta_{15j}*healthy_{ij}+\beta_{16j}*loginc_{ij}+\beta_{17j}*hhsize_{ij}+\sum_{m=1}^{2} D_{m}d_{m} \end{matrix}$ （8）


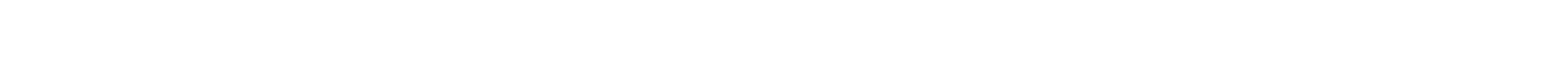

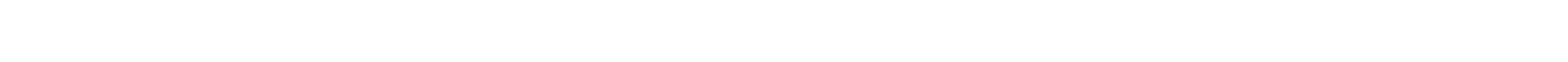

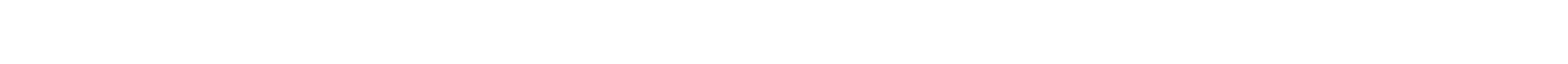


Equation 8 is a proportional odds model. In equation 8, $D_{m}$ denotes the respondent’s answer to the happiness question and $\delta_{m}$ is the threshold. When *m* = 1 (‘very happy’), $D_{m}=0$; when *m* = 2 (‘pretty happy’),$D_{m}=0$.


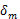


At level 2, we used survey year to predict the parameters in equation 8. Given that the data are cross-sectional data, the auto-regression effect is negligible. The different waves were treated as a categorical variable. The Gini coefficient and its quadratic term were included in intercept model at level 2 (equation 9). Because previous studies have identified small auto-correlations in the GSS data (Yang, 2008), we also included the survey year in the intercept model to control for its impact. At the same time, because GDP growth rate represents economic opportunities and can influence happiness, we also included it as a level 2 control variable. Additionally, we specified a level 2 model (slope equation) for each slope in equation 8. For all slope equations, the random effects of survey years were evaluated. However, only for the slope equation of income, the Gini coefficient was included to test its moderating effect on the income-happiness relationship. Therefore, we can rewrite the level-2 equations as follows:

$\beta_{0j}=\gamma_{00}+\gamma_{01}*gini_{j}+\gamma_{02}*ginisquared_{j}\text{+}\gamma_{03}*gdprate_{j}\text{+}\gamma_{04}*surveyyear_{j}\text{+}\mu_{0j}$ （9）


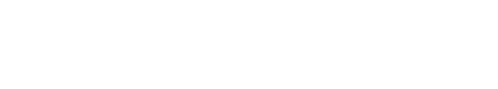


$\beta_{16j}=\gamma_{160}+\gamma_{161}*gini\text{+}\mu_{16j}$ （10）


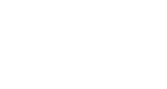


$\beta_{kj}=\gamma_{k0}+\mu_{kj}$，*k* = 1,2,3,…,15 （11）


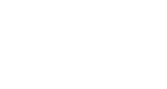


where *μ* denotes the random effect of survey year and is assumed to have a multivariate normal distribution, and all *γ_s_* are fixed effects.

### Model Specifications in Study Two

In the analysis of the European data, the level 2 unit of analysis was country. Let *Y_ij_* denote the happiness level of individual *i* in country *j*., The linear mixed-effects models (Raudenbush & Bryk, 2002) for the 2002, 2004 and 2006 waves of ESS data can be expressed as follows:

Level 1 or individual level model:


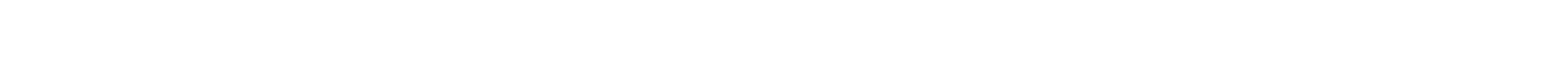

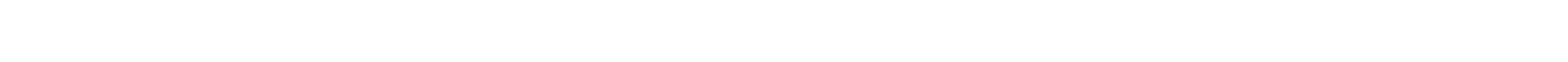

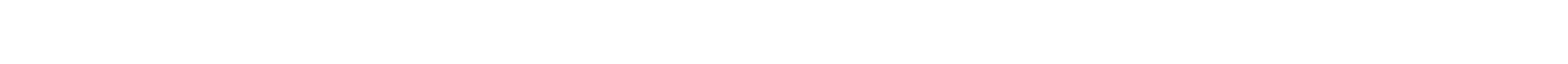
（12）

Level 2 or country level model:


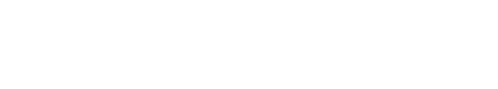
 （13）


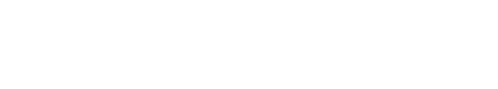
，*k* = 1,2,3,…,20 （14）

Demographical and income variables were controlled in the first level and GDP growth rates were controlled in the second level. The GSS model further controlled surveyed time. Critically, both national Gini coefficients and its quadratic terms were entered as predictors in the second level to modeling the curving relationship between Gini and happiness (see Supp. Mat. for model specifications).
